# POF and HP1 Bind Expressed Exons, Suggesting a Balancing Mechanism for Gene Regulation

**DOI:** 10.1371/journal.pgen.0030209

**Published:** 2007-11-16

**Authors:** Anna-Mia Johansson, Per Stenberg, Fredrik Pettersson, Jan Larsson

**Affiliations:** Umeå Center for Molecular Pathogens, Umeå University, Umeå, Sweden; European Molecular Biology Laboratory, Germany

## Abstract

Two specific chromosome-targeting and gene regulatory systems are present in Drosophila melanogaster. The male X chromosome is targeted by the male-specific lethal complex believed to mediate the 2-fold up-regulation of the X-linked genes, and the highly heterochromatic fourth chromosome is specifically targeted by the Painting of Fourth (POF) protein, which, together with heterochromatin protein 1 (HP1), modulates the expression level of genes on the fourth chromosome. Here we use chromatin immunoprecipitation and tiling microarray analysis to map POF and HP1 on the fourth chromosome in S2 cells and salivary glands at high resolution. The enrichment profiles were complemented by transcript profiles to examine the link between binding and transcripts. The results show that POF specifically binds to genes, with a strong preference for exons, and the HP1 binding profile is a mirror image of POF, although HP1 displays an additional “peak” in the promoter regions of bound genes. HP1 binding within genes is much higher than the basal HP1 enrichment on Chromosome 4. Our results suggest a balancing mechanism for the regulation of the fourth chromosome where POF and HP1 competitively bind at increasing levels with increased transcriptional activity. In addition, our results contradict transposable elements as a major nucleation site for HP1 on the fourth chromosome.

## Author Summary

Species where males and females have a different number of sex chromosomes have to equalize the transcriptional output from the genes located on the X chromosome. In *Drosophila* this mechanism is achieved by a 2-fold up-regulation of the single male X chromosome. Flies also possess an additional chromosome-wide regulatory system that regulates the transcriptional output from genes on the fourth chromosome. In this case the protein Painting of Fourth (POF), together with heterochromatin protein 1 (HP1) bind to the fourth chromosome and fine tune gene expression. By using a high resolution map of POF and HP1 binding, we can show that they bind to the same sequences on the fourth chromosome. We also demonstrate that POF and HP1 bind to active genes with preferences for exon sequences. In gene regulatory mechanisms, including chromosome-wide gene regulation, a simple on/off switch is often not enough. Our findings support the presence of a balancing mechanism in which the dual recruitment of a repressing and a stimulating factor makes the transcription efficiency more stable and less sensitive to fluctuations.

## Introduction

The chromatin of eukaryotes is highly organized and can be functionally divided into active euchromatin and silent heterochromatin [1,2]. Telomeres and pericentric regions are the main chromosomal domains that consist of constitutive heterochromatin. However, in *Drosophila* the small fourth chromosome is also considered to be highly heterochromatic [3]. The fourth chromosome has an overall length of ∼5 Mb, 3–4 Mb of which consists of satellite repeats with no known genes [4]. The remaining portion (1.23 Mb) corresponds to the sequenced and banded part of the chromosome, includes 92 genes and, thus, has a gene density similar to that of the major chromosome arms. Chromosome 4 is late replicating [5] and does not exhibit meiotic recombination under normal conditions [6–8]. The banded region contains unique sequences interspersed with repetitive DNA with an unusually high content of transposable elements [9–14]. Importantly, transgenes inserted into this chromosome are often partially silenced and their expression is variegated, like that of transgenes inserted close to heterochromatin [15–17].

We have recently shown that the Chromosome 4-specific protein Painting of Fourth (POF) is important for correct transcriptional output of the genes on the fourth chromosome [18]. POF is a putative RNA-binding protein that binds throughout the polytenised and sequenced part of the fourth chromosome [19,20]. The binding of POF to the fourth chromosome has been conserved during evolution. In several species within the genus *Drosophila*, POF is specifically localized to the F-element, which corresponds to the fourth chromosome in *D. melanogaster* [21]. The binding of POF to the fourth chromosome mimics the binding of the dosage compensating male-specific lethal (MSL) complex to the male X chromosome in *Drosophila* [20]. Indeed, it appears likely that POF binding to the fourth chromosome derives from a dosage compensating system. In the distantly related species D. busckii, POF specifically decorates the male X chromosome and also colocalizes with histone 4 acetylated at lysine 16 (H4K16Ac), a histone modification associated with dosage compensation in flies. In D. ananassae and D. malerkotliana POF is also specifically associated with the male X chromosome and colocalizes with the dosage compensation complex protein MSL-3 [21]. These findings support the proposed relationship between the fourth chromosome and the X chromosome. Indeed, it has been argued that the fourth chromosome originates from the X chromosome (for reviews see [3,7,20]).

The binding of POF to the fourth chromosome is dependent on heterochromatin and loss of *Pof* function causes a general decrease (on average 14%) in Chromosome 4-specific gene expression, suggesting that POF stimulates the expression of genes on the fourth chromosome [18]. Furthermore we showed that POF and the heterochromatin associated protein 1 (HP1) bind interdependently to the fourth chromosome [18]. HP1 is a chromodomain protein that targets di- and tri-methylated histone 3 lysine 9 (H3K9me2/3) [22–24]. Although Su(var)3–9 is the main histone methyl transferase responsible for H3K9me2/3 methylation, it is not responsible for H3K9 methylation on the fourth chromosome [25,26]. It has recently been shown that SETDB1 is the enzyme responsible for H3K9 methylation on the fourth chromosome [27,28]. Immunofluorescence microscopic analysis of HP1 has revealed that it binds to pericentric heterochromatin and to a number of discrete bands along the chromosomes [29–31]. HP1 also binds along the length of the fourth chromosome and, at the cytological level, colocalizes with POF on the polytenized fourth chromosome [18]. Recent mapping at a higher resolution, using the DamID technique, has shown that HP1 binds within transcribed genes and the role of HP1 as a repressive protein may, therefore, be questioned [32]. Although binding data suggest that HP1 binds active genes, analysis of gene expression following *HP1* RNA-mediated interference has indicated that the genes on the fourth chromosome are generally up-regulated (on average 12%) upon the loss of HP1, thus supporting the suggestion that it is repressive [18].

High resolution binding data and complementary information on transcript levels and profiles are essential for elucidating the mechanisms that control the expression of genes on the highly heterochromatic fourth chromosome and ensure that chromosome-specific regulatory systems are correctly targeted. In the study presented here we mapped POF, HP1, and acetylated H3K9 at high resolution on the fourth chromosome in two types of D. melanogaster cells: Schneider 2 cells and salivary gland cells, using chromatin immunoprecipitation (ChIP) and tiling arrays covering the fourth chromosome in 10-bp intervals. In addition, we analyzed the transcript profiles of the two cell types used. Our data suggest that POF and HP1 interdependently bind to genes with a strong preference for exon sequences. HP1 also exhibits a distinct binding peak in the promoter region of most bound genes. Combining the binding data with the transcript profiles of the two cell types used further demonstrates that the competitive binding of POF and HP1 correlates to levels of transcription.

## Results

### POF Binds Exclusively to the Fourth Chromosome in D. melanogaster

**Figure 1:**
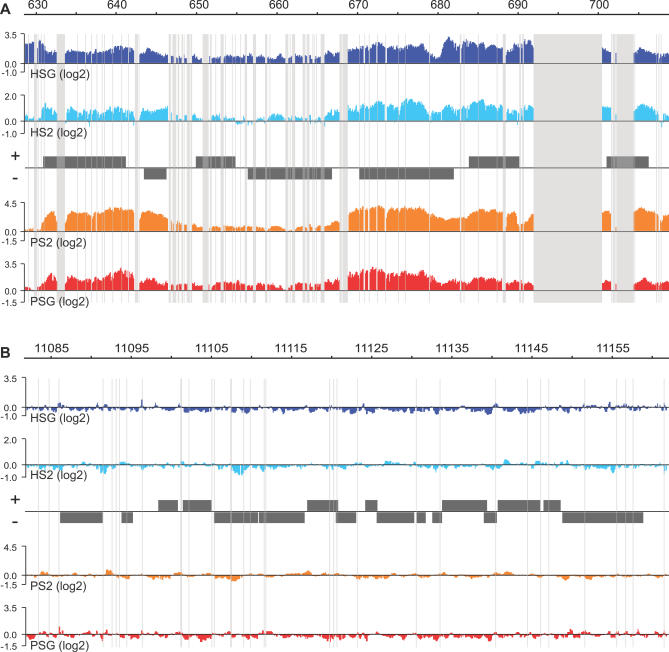
High Resolution ChIP-chip Analysis of POF and HP1 Binding to Chromosomes 4 and 2L in S2 Cells and Salivary Glands The smoothed data for one of the three biological replicates of each experiment are shown. Figure S1 shows a comparison of the replicates and the effect of the smoothing process. A typical 80-kb region from the fourth chromosome (A) is compared to a typical 80-kb control region from Chromosome 2L (B). The scale bars show the genomic positions of the two regions in kb. HP1 enrichment in salivary glands is shown in dark blue (HSG), HP1 in S2 cells (HS2) in light blue, POF in S2 cells (PS2) in orange, and POF in salivary glands (PSG) in red. The enrichments are the log2 ratios between specific IP/input. Genes expressed from left to right are shown above the horizontal line in each panel, and the genes expressed in the opposite direction are shown below the line. Regions that are repeat-masked and therefore not represented by probes on the arrays are shaded. Note that the fourth chromosome is enriched in repeated regions.

To determine the precise locations of POF and HP1 along the fourth chromosome in D. melanogaster we designed tiling arrays (NimbleGen Systems) composed of the complete repeat-masked fourth chromosome with 50-bp oligos at 10-bp intervals (i.e., with a 40-bp overlap between consecutive oligos). Although this will provide higher resolution than generally provided by the ChIP technique because of the fragmentation of chromatin down to 500–1,000 bp, it makes it possible to include a sliding window smoothing without affecting resolution. The high oligo density on the array also allows transcript profiling on the identical array platform. A region from Chromosome 2L (12 Mb) with 50-bp intervals and 10,000 random probes was also included on the array. ChIP was used to isolate regions exhibiting abundant POF and HP1 binding. For each experiment we made three biological replicates. The enrichment profiles of the different replicates were very consistent (Figure S1), so we chose one biological replicate of each type for further studies. A typical 80-kb region from the fourth chromosome and a control region from Chromosome 2L are shown in Figure 1. The results show that POF is highly specific to the fourth chromosome and binds to certain regions. In addition, HP1 enrichment follows the enrichment profile of POF (as discussed in detail below). The HP1 binding profile presented here follows and corroborates the binding profile published using DamID on *Drosophila* Kc cells [32]. However, our work on the fourth chromosome provides higher resolution, resulting in more information and more detailed conclusions about HP1.

### POF Binds Preferentially within Genes with Exon Bias

**Figure 2:**
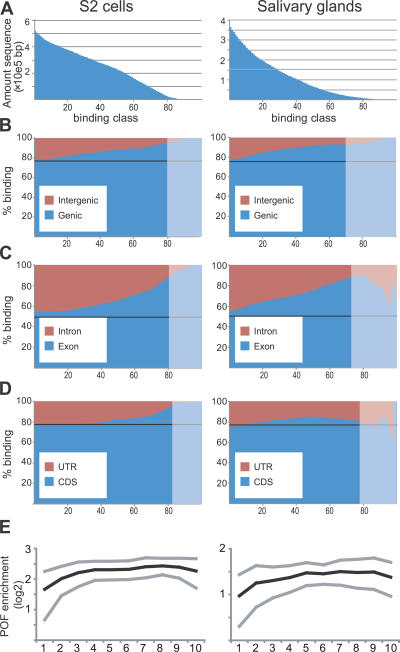
POF Binds Preferentially within Genes with a Preference for Exons (A) The amounts of sequences with binding divided into 100 binding classes. In S2 cells, 509 kb out of 860 kb qualified as binding compared to 377 kb in salivary glands. The amounts of intergenic compared to genic sequences (B), exons compared to introns (C), and UTR compared to coding sequences (D) plotted against increasing (stronger) binding classes. S2 cells are shown to the left of salivary glands. The black line corresponds to expectations if the binding was randomly distributed, and the shaded areas represent the situation when only 5% of the probes are left, ranging from 6.5 kb (CDS in salivary glands) to 20 kb (genes in S2 cells). These areas will therefore be sensitive to random effects and annotation inaccuracies. (E) The average binding profile of POF to bound genes in S2 cells and salivary glands. Exons from genes of different lengths were scaled to align 5′ and 3′ ends and divided into ten regions. The mean binding of each region of the gene is represented by black lines and the standard deviations by gray lines.

Alignment of the binding data to the annotations clearly showed that POF binds preferentially to genes. We divided all probes with enrichment levels higher than our calculated threshold value (binding probes) into sets with 100 different binding levels from 1 to 100 (Figure 2A) and used a stepwise process, by examining the distribution of probes with increasing binding levels, thereby gradually removing enrichment caused by technical spreading as a consequence of ChIP. Any bias towards a specific category was, therefore, detected as an increase in percentage binding with increased binding classes. Comparison of the binding strength of gene regions and intergenic regions showed that POF binds preferentially within genes in both S2 cells and salivary glands (Figure 2B). Next, comparison of exon with intron regions showed that the preference is also strongly biased towards exon sequences in both cell types (Figure 2C). Exon densities tend to be higher at the 3′ ends of genes. To ensure that the observed exon bias is not a consequence of a general bias in POF binding to the 3′ ends of genes, we recalculated the comparison between exon and introns but removed 25% and 50% of the 3′ ends of the genes. If there is a 3′ bias in binding and this causes the exon preference, removal of increasing amounts of 3′ parts of the genes should proportionally remove the exon bias. It turns out that in both of these cases the exon bias is similar to when using complete genes (unpublished data). We also compared the coding sequence of each gene to the UTRs. The results suggest that there is also a preference for coding regions, but only in S2 cells (Figure 2D). It should be noted that this bias is less pronounced and may, to some extent, reflect the fact that UTRs always border intergenic regions whereas coding sequences do not. Given the strong preference towards genes, we decided to calculate relative binding values for POF and HP1 binding for each gene in both cell types (Table S1). The relative binding values between the three different replicates for each experiment were very similar, indicating that the quality of our data allows a qualitative assessment of binding (Figure S2). With the cutoff used, 69% of Chromosome 4 genes were bound by POF in S2 cells and 56% in salivary glands, compared with 0.88% and 0.15%, respectively, of genes on Chromosome 2. It should be stressed that all bound genes on the second chromosome have comparable binding levels to the 10% weakest bound genes on the fourth chromosome. To determine the distribution of binding of POF along the genes classified as bound, we scaled all genes to the same relative length after removing all introns (since there is a strong exon bias). We then calculated relative binding levels along the genes. A weak bias was seen toward the 3′ ends of the bound genes in both S2 cells and salivary glands (Figure 2E).

### The Same Set of Chromosome 4 Genes Are Targeted by POF and HP1

**Figure 3:**
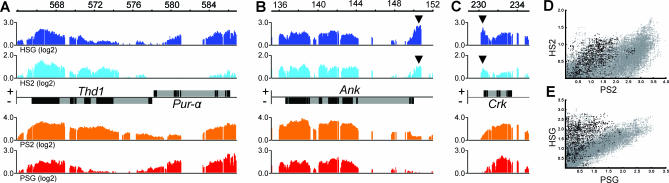
POF and HP1 Colocalize within Genes while HP1 Binding Shows an Additional “Promoter Peak” (A) POF and HP1 enrichment profiles at the *Thd1 Pur-alpha* gene pair locus. POF and HP1 binding at the *Ank* locus (B) and the *Crk* locus (C). HP1 binding in salivary glands is shown in dark blue, HP1 in S2 cells in light blue, POF in S2 cells in orange, and POF in salivary glands in red. Genes expressed from left to right are represented by rectangles above the horizontal line in each panel and the genes expressed in the opposite direction are shown below the line. Exons are indicated in black and introns in gray. Note the peak of HP1 enrichment (arrowheads) in the region upstream of the transcription start points of *Ank* and *Crk*, present in both S2 cells and salivary glands. All enrichment values for all single probes on the fourth chromosome plotted as HP1 enrichment on the *y*-axis and POF enrichment on the *x*-axis for S2 cells (D) and salivary glands (E). Probes in the putative promoter region (−500 to +200 bp from the transcription start point) are shown in black.

We have previously shown that, at the cytological level, POF and HP1 colocalize and that the same set of genes is regulated by these two proteins [18]. We wanted to analyze if POF and HP1 colocalize at the gene level, which can be determined at the resolution provided by ChIP-chips. As shown in Figure 3A, POF and HP1 exhibit very similar enrichment profiles within genes. There is, however, an exception to the almost perfect correlation between POF binding and HP1 binding, in the promoter regions. In most bound genes a clear peak in HP1 binding was seen in the promoter region, as exemplified in Figure 3B and 3C. Plotting all POF binding scores (at the individual probe level) against all HP1 binding scores reveals that, in general, there is a linear correlation between POF binding and HP1 binding. By marking probes within the promoter region of each POF-bound Chromosome 4 gene it was clear that the exceptions to the linear correlation are mainly due to the HP1 peak in the promoter region (Figure 3D and 3E). Using conventional motif-finding algorithms, we were unable to correlate the HP1 promoter peak to a specific motif. The HP1 enrichment is higher in salivary glands than in S2 cells. It should also be stressed that, in salivary glands, HP1 shows a higher basal level of enrichment on Chromosome 4 in intergenic regions than POF (Figure 3E). We conclude that POF and HP1 colocalize within the transcribed region of individual genes with a preference for binding exons, but HP1 also exhibits a peak in the promoter region of most bound genes and a higher basal enrichment on the fourth chromosome.

### POF and HP1 Bindings Are Correlated to Transcription

**Figure 4:**
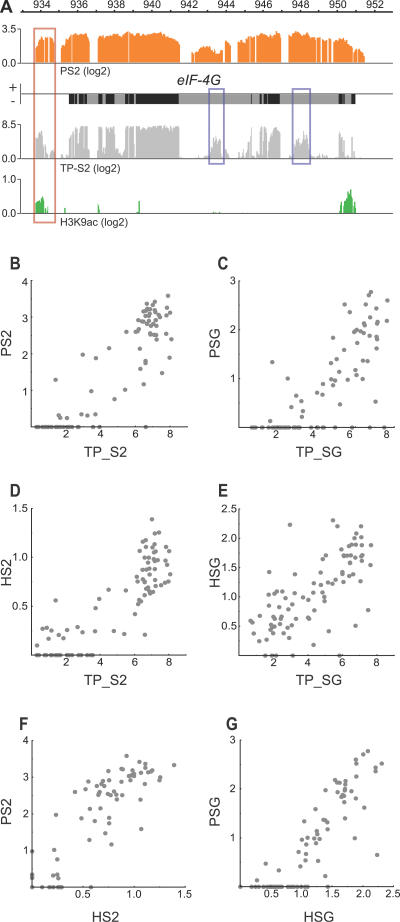
Correlations of POF and HP1 Binding Strength with Each Other and with Transcription Levels (A) Transcript profiling (light gray), POF enrichment (orange), and H3K9ac enrichment (green) at the *eIF-4G* locus (transcribed from right to left) in S2 cells. Note that the transcript profile allows predictions of exon usage and transcript levels of unannotated exons (exemplified by blue boxes). The H3K9ac is typically enriched at the 5′ end of transcribed genes, as seen for *eIF-4G*. The combination of transcript profiling and the H3K9ac profile also allows prediction of novel genes, e.g., a novel gene can be predicted downstream of *eIF-4G* (red box) transcribed from left to right. (B) POF binding in S2 cells (PS2) plotted against relative transcription values in S2 cells (TP_S2). (C) POF binding in salivary glands (PSG) versus transcription values in salivary glands (TP_SG). (D) HP1 binding in S2 cells (HS2) versus relative transcription values in S2 cells. (E) HP1 binding in salivary glands (HSG) versus relative transcription values in salivary glands. (F) POF binding in S2 cells versus HP1 binding in S2 cells, and (G) POF binding in salivary glands versus HP1 binding in salivary glands. Note the more even distribution of different transcription levels in salivary glands compared to S2 cells.

To relate the binding of POF and HP1 to chromatin structure we also examined the distribution of H3K9ac (Histone 3 acetylated at lysine 9) in S2 cells. H3K9ac is a marker for “active” chromatin. The binding profile of H3K9ac demonstrates that acetylated H3K9 is highly enriched in the 5′ region of most genes bound by POF and HP1 (Figure 4A and unpublished data). To examine the relationship between the binding of POF and HP1 to gene expression in more detail, we probed identical arrays using cDNA from the cell types under consideration. We prepared mRNA from two biological replicates of both S2 cells and salivary glands, reverse transcribed it to cDNA, labelled probes, and hybridised them to our designed arrays. In this manner we were able to obtain high quality expression data for each gene as well as a detailed map of the exons that are used and their usage ratios. Since the double-stranded cDNA was generated prior to labelling we were unable to determine which strand a given transcript originated from. The transcript profile data can be used to detect exon usage and, since H3K9ac is usually enriched at the 5′ region of transcribed genes, the combination of transcript profiling with the H3K9ac binding profile generates predictions of novel genes (exemplified in Figure 4A). From the transcript profile data we calculated a relative transcription value for each gene in each cell type (Table S1). The calculated transcription values were consistent in the two replicates (Figure S3). The binding of both POF and HP1 correlates highly with transcription. In salivary gland cells the transcription values are typically more variable, while in S2 cells the genes tend to be expressed at either a high or low level (Figure 4B–4E). Furthermore, the HP1 binding values for each gene correlate well with the relative binding values of POF, further supporting the suggestion that the binding levels of HP1 and POF are interdependent (Figure 4F and 4G). Mean transcript profiles along all Chromosome 4 genes in the two cell types are similar to the POF binding profiles (Figure 2E) and show a 3′ bias as well (unpublished data). The 3′ bias of the transcript profiles may, however, be caused by incomplete reverse transcription.

### Cell Type–Specific Binding of Genes Is Coupled to Transcript Levels

**Figure 5:**
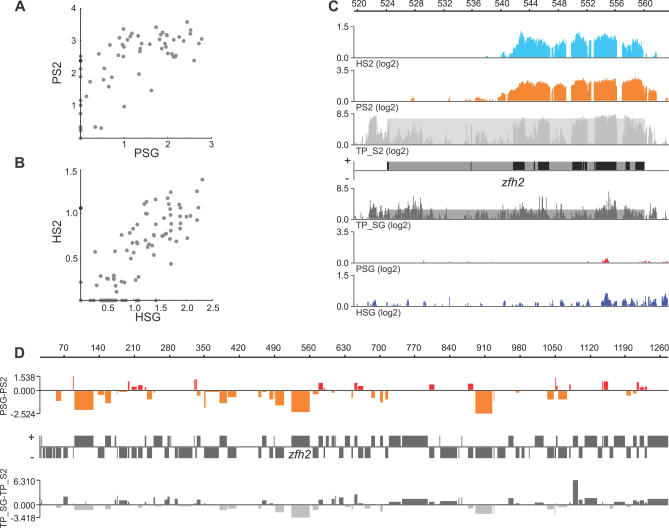
Cell-Specific Binding of POF and HP1 Is Linked to Levels of Transcription Relative binding values for POF (A) and HP1 (B) for Chromosome 4 genes in S2 cells versus salivary glands. The *zfh2* gene is indicated as a black dot. (C) POF and HP1 enrichments at the *zfh2* locus in S2 cells and salivary glands. The transcript profiles on a log2 scale in S2 cells (light gray) and salivary glands (dark gray). The shaded boxes indicate the calculated expression values for *zfh2* in the two cell types. (D) The log2 differences in POF binding and differences in expression between salivary gland cells and S2 cells plotted with respect to the positions of the genes along the fourth chromosome. Genes with higher binding levels in salivary glands are shown as red bars above the horizontal line while genes with higher binding levels in S2 cells are shown as orange bars below the line. The widths of the bars reflect the gene length. The relative change in expression is shown below the gene annotation map (the *zfh2* gene is indicated). Genes that are more expressed in salivary glands are shown as dark gray bars above the horizontal line, and genes that are more expressed in S2 cells are shown as light gray bars below the horizontal line. Note that the POF binding differences are reflected by the differences in expression.

We were interested to see how differentially expressed genes behave in binding POF and HP1. Although most genes bind HP1 and POF in the same way, there are examples of genes with differential binding between the cell types (Figure 5A and 5B). A good example of differential binding is seen in the *zfh2* gene, which is strongly bound in S2 cells by both POF and HP1, but weakly bound in salivary gland tissue (Figure 5A–5C). As shown in the figure, the binding profiles of POF and HP1 not only correlate with each other but also strongly reflect the differences in transcription levels (Figure 5C). We therefore plotted differences between POF binding in S2 cells and salivary gland cells against the differences in transcript values between the two cell types for each Chromosome 4 gene. The results show not only that high POF binding correlates to high expression, but also that this is cell-type dependent. This means, for example, that a gene that is more strongly expressed in S2 cells than in salivary glands will also bind a higher level of POF in S2 cells than in salivary glands (Figures 5D and S4).

### Transgenic Silencing on the Fourth Chromosome Is Correlated to HP1 but Not to Transcriptional Silencing of Chromosome 4 Genes

**Figure 6:**
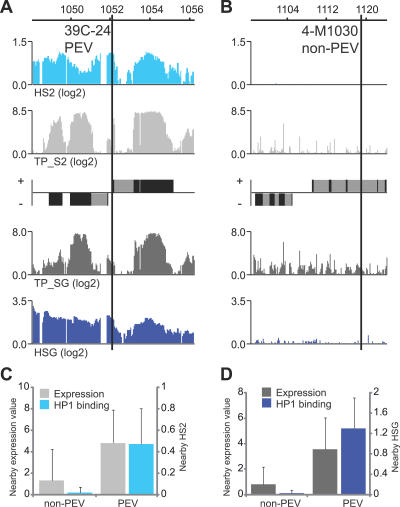
Silenced Trangene Insertions Correspond to Sites with High HP1 Enrichment but Not with Low Expression Regions (A) HP1 enrichment profiles and transcript profiles surrounding the insertion of the silenced transgene 39C-24 and the nonsilenced transgene 4-M1030 (B). (C) Both the HP1 binding and the transcript levels are lower in the regions near (±500 bp) nonsilenced insertions (non-PEV) compared to regions near silenced insertions (PEV) in S2 cells and salivary glands (D). The silenced (*n* = 17) and nonsilenced (*n* = 6) transgenes are from Sun et al. [35].

Chromatin enriched in HP1 is well known for its ability to repress gene expression. Nevertheless, our data indicate that on the fourth chromosome HP1 is positively correlated with transcription. A large number of transgene insertions on the fourth chromosome have been extensively studied and a large proportion of those have been shown to be partially silenced, as demonstrated by position-effect variegation (PEV). We decided to determine whether the transgenic insertions that display PEV silencing are located in regions with high levels of HP1 and therefore in regions that are transcribed, or whether they are also inserted in genes that are expressed at low levels on Chromosome 4. It should be stressed that we only measured HP1 binding and transcription in salivary glands and S2 cells, and there might be differences in binding and transcription levels of genes between these tissues and the eye discs. Even so, the results strongly suggest that high levels of HP1 can induce silencing of reporter genes (Figure 6A–6D). Strikingly, the silenced insertions are not correlated to regions where Chromosome 4 genes are silenced or weakly expressed. We conclude that high levels of HP1 correlate with the silencing of nearby transgenic insertions, but not with low expression of Chromosome 4 genes.

### HP1 and POF Are Not Targeted by Repeats or Transposable Elements

**Figure 7:**
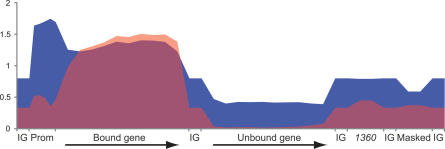
Mean Enrichment Profiles of HP1 and POF in Salivary Glands, Calculated from Enrichment Values for All Bound (by POF) and All Unbound Chromosome 4 Genes (Merged Exons) The promoter regions of bound genes (500 bp upstream of the transcription start point) were divided into five 100-bp fragments, and the mean enrichment in each fragment was calculated and is indicated. The mean enrichments within intergenic regions (IG), surrounding (±200 bp) repetitive regions (Masked), and *1360* elements are indicated. The enrichment of HP1 and POF is shown in blue and red, respectively.

There is evidence suggesting that heterochromatin proteins, such as HP1, preferentially associate with transposable elements [33]. It has also been suggested that certain transposable elements, e.g., *1360*, are recruitment sites for HP1 and that the HP1-mediated silencing of reporter genes may be caused by the heterochromatic structure spreading from *1360* [34,35]. Since repetitive elements are masked by the design of our arrays, we were unable to directly score HP1 and POF enrichment within those sequences. However, since the chromatin fragments used in the ChIP experiments are generally 500–1,000 bp long, we do expect technical spreading of enrichment to occur. Consequently, if repetitive elements were targets for HP1, we would expect the nearby regions that are not masked on our arrays to also show increased enrichment of HP1. However, in the two cell types analyzed we found no evidence that repetitive elements were enriched more strongly than the basal HP1 binding of the fourth chromosome. Indeed, the enrichment levels close to these masked regions were much lower than the enrichment levels seen in bound genes (Figure 7 and unpublished data). We conclude that the high enrichment of HP1 within genes, and probably within inserted transgenes, is not caused by a spread of HP1 from transposable elements such as *1360*. We next calculated whether the distribution of *1360* is random or biased towards bound genes. If the positions of *1360* elements on the fourth chromosome are randomized, the numbers close to or within bound genes is similar to the actual values (unpublished data). To provide an overview of our data, we calculated mean enrichment profiles for bound and unbound genes, promoters, intergenic regions, masked regions, and *1360* elements (Figure 7). In summary, our tiling data for POF and HP1 show that both these proteins bind within genes, with a strong preference for binding exons. In addition to binding within the transcribed region of the genes, HP1 (but not POF) also exhibits a higher basal level of binding to the fourth chromosome and displays a promoter-specific peak in most bound genes (Figure 7). However, we observed no preference of HP1 for repetitive or transposable elements.

## Discussion

The fourth chromsome provides an excellent model for studying the regulation of gene expression in highly heterochromatic regions and for examining chromosome-wide gene regulation [20]. We have previously shown that POF stimulates gene expression specifically on the fourth chromosome, while the interdependently bound HP1 represses. Although HP1 is a general component of heterochromatin, POF is specifically targeted to the fourth chromosome and will not spread into other chromosomal regions translocated to the fourth chromosome [19]. POF targeting depends on Chromosome 4 specific factors and the amount of heterochromatin. Although the mechanism of targeting remains partly unanswered our current work makes an important advance in demonstrating the relationship between POF, HP1, and transcription activity.

### POF Targets within Genes with Exon Bias

We previously mapped POF at a 2-kb resolution within three individual genes, and the result suggested that POF binds within genes [18]. Here, we mapped POF binding to the entire sequenced part of the fourth chromosome using high resolution ChIP-chip. The results show that POF binds within genes. Although some regions lack POF this is associated with a lack of transcription, indicating that we must consider POF binding to be gene specific. We found that POF binding is highly correlated with both expression state and levels of expression in salivary gland and S2 cells. Furthermore, differences in expression levels between the two cell types reflect comparable differences in POF and HP1 binding. We conclude that the binding is connected to both transcription and also levels of the resulting transcripts. Transcription activity precedes POF binding since the loss of *Pof* results only in a slight reduction of transcription [18]. POF binds preferentially to genes rather than intergenic regions. In fact, we did not detect convincing POF binding in any region outside a transcribed region. Furthermore, POF binds preferentially to exons rather than introns. We note a slight 3′ bias in POF binding, however, we cannot dismiss the possibility that this observed 3′ bias may be due to longer exons tending to be located at the 3′ end of genes. Notably, the gene binding profile reflects the transcript profile, which also shows a 3′ bias (unpublished data). Interesting questions are raised by these findings about the nature of the mechanisms responsible for the strong bias towards exons. We propose two possible scenarios. First, spliced RNAs may be used in the targeting mechanism for gene-targeting proteins such as POF. Second, and more likely, there may be crosstalk between POF and the splicing machinery. It has been shown recently that the human SWI/SNF subunit Brm, involved in chromatin remodelling, favours the inclusion of variant exons in the mRNA of several genes [36]. The cited authors suggest that Brm decreases the RNAPII elongation rate and facilitates recruitment of splicing machinery to suboptimal splice sites. POF also binds intronless genes, so splicing per se does not explain the binding. However, some shared mechanistic step may explain the binding profile and provide a link to the POF-mediated facilitation of transcription.

It is intuitively difficult to envision how sequence determinants for targeting can be present in exons. In this context it should be stressed that we have previously shown that exons on the fourth chromosome as well as exons on the X chromosome differ in sequence composition compared to exons of the other chromosomes [14]. Therefore, sequence based determinants within exons responsible for protein targeting should not be ruled out.

We believe that transcript profiling is a robust method for determing relative transcript levels of genes since all genes are measured with a large number of oligos and differences in the expression of individual exons is also easily detected. Our transcript profiling provides a tool for refined annotation of the fourth chromosome and prediction of novel exons and genes. H3K9 acetylation is highly enriched in the 5′ region of most active genes. This means that combining the H3K9ac profile with the transcript profile increases the precision in discriminating between novel genes and additional exons. A similar profile for H3K9 acetylation in yeast has been previously described by Pokholok et al. [37]. However, they found that H3K9 acetylation peaks at the transcription start point rather than within the transcribed region of genes. The difference in our results and theirs is probably due to the fact that we measured the amount of H3K9ac per se, while Pokholok et al. measured it relative to the levels of core histone H3 [37]. The lower nucleosome density within the promoter region is probably responsible for this difference in the location of the peak.

### HP1 Targets Genes and also Their Promoters

We have previously shown that POF and HP1 colocalize at the resolution given by polytene chromosome and that the same set of genes is regulated by these two proteins [18]. We show here that POF and HP1 binding colocalize at the gene level, which also provides insights into the modes of action for this regulatory system. The bias in within-gene binding towards exons shown by POF was also observed for HP1. HP1, like POF, preferentially binds exons but, in contrast to POF, HP1 shows a high basal binding to the fourth chromosome and a peak in binding associated with promoters for most targeted genes. The specificity of HP1 to certain promoters at the individual target gene level has previously been reported in mammals [38–41]. According to our data the promoter peak of HP1 is a more general characteristic of HP1-bound genes on the fourth chromosome and is related to transcription. It has been proposed that the presence of H3K9me at promoters is connected to gene repression, but that H3K9me within the genes is associated with gene activity [42]. If our HP1 profile is assumed to be linked to the presence of H3K9me this implies that a combination of these two binding profiles is linked to the transcription of genes on the fourth chromosome.

The classical view is that HP1 is associated with gene repression. However, a number of recent reports have linked HP1 to gene activation, based on the enrichment of H3K9me and HP1 on active genes. It is well known that HP1 is enriched in pericentric regions and that genes in those regions are, therefore, connected to high HP1 levels. It has been shown that mutation in *HP1* causes a reduction in the expression of a number of heterochromatin-located genes in *Drosophila* e.g., *light* and *rolled*, supporting the idea that some genes depend on their heterochromatic surroundings for correct expression [43–45]. However, it has also been demonstrated that HP1 is associated with the transcribed regions of active genes located in euchromatic regions [32]. In addition, HP1 has been shown to associate with developmental and heatshock-induced puffs of the polytene chromosome, which is indicative of intense gene activity [46]. It has been shown that H3K9 methylation occurs in the transcribed region of active genes in mammalian chromatin and, in fact, increases during activation of transcription [47]. In the cited study case HP1 was found to be associated with the transcribed genes of several mammalian cell lines and also in primary cells. However, it is important to note that, except for the heterochromatin genes *light* and *rolled*, it is not clear whether the binding of HP1 is associated with facilitated transcription. Our results indicate not only that HP1 binds to active genes on the fourth chromosome, but also that the genes on the fourth chromosome are up-regulated upon loss of HP1 [18]. Thus, although the genes on the fourth chromosome are bound in response to gene activity, HP1 still causes repression. It may be that, for example, heat-shock induced genes attract HP1 as a modulator that represses uncontrolled gene expression. In contrast to HP1, the loss of POF leads to a general decrease in gene expression from the fourth chromosome [18]. The strong correlation with respect to binding between HP1 and POF and their correlation with transcription support our balancing model [18]: POF stimulates and HP1 represses gene expression and the interdependent binding of these two proteins fine tunes the expression output from the fourth chromosome. It should be noted that the correlation between HP1 and POF seems to be linear, suggesting that highly expressed genes have the same POF/HP1 ratio as genes with weak expression, although they bind higher amounts of both proteins. A balancing mechanism may act as a buffering system in which the dual recruitment of a repressing and a stimulating factor makes the transcription efficiency more stable and less sensitive to fluctuations. Balancing mechanisms may be more general. For example, this may explain the proposed binding of HP1 to the male X chromosome [33]. The facilitated transcription of X chromosomal genes by acetylation of H4K16 may need to be tempered by a repressing factor to reach the expected 2-fold increase. This repressing function might be supported by HP1, Su(var)3–7, or other unknown factors not yet linked to dosage compensation.

The targeting of POF to the fourth chromosome shows similarities to the targeting of the MSL complex to the male X chromosome [48–50]. The striking similarity between POF and the dosage-compensating MSL complex in evolutionary terms [19,21], their function as chromosome-wide regulators [18], and their binding profiles, as presented here, supports a common origin. For the MSL complex, expressed genes are the main targets. In addition, in fly embryos, S2 cells, and in a cell line derived from larval imaginal discs (Clone8 cells), MSL binding is also associated with expressed genes, but does not correlate with level of expression [48,49]. It has been demonstrated that, to a large extent, MSL binding is stable throughout development and that the binding reflects the expression levels in young embryos (4–5 h) [50]. We hypothesize that a similar strong correlation between levels of transcript and binding as well as cell type differences as seen for POF, might be true also for MSL if studied at higher resolution. A correlation between binding levels and levels of transcription would be in line with the expected 2-fold increase of gene expression independendent of expression levels.

### Transgenic Silencing on the Fourth Chromosome Correlates to HP1

It has been shown that transgenes inserted on the fourth chromosome are often partially silenced and that the localization of these variegated insertions in some regions of chromosome 4 is correlated to their distance from the transposable element *1360* [35]. This, along with the fact that the *1360* element can contribute to the silencing of an adjacent reporter when close to pericentric heterochromatin, suggests that *1360* elements may serve as HP1 recruitment signals [34,35]. Further support for this hypothesis is provided by the suggestion that repeat flanked genes are more likely to bind HP1 [33]. Our results show that HP1 binds Chromosome 4 genes, but we found no indications of transposable elements such as *1360* acting as nucleation sites for HP1. It should be stressed that these results do not contradict the reported correlation between transgenic silencing and distance from *1360* elements [35]. It is possible that *1360* elements under certain conditions serve as nucleation sites for heterochromatin formation and spread, but that this does not involve HP1. It is also possible that *1360* elements act as initial nucleation sites for HP1, which are not maintained in the two cell types analyzed. Furthermore, silenced transgene insertions on Chromosome 4 are linked to regions with relatively high binding of HP1, but these regions are typically expressed. This implies that the transcriptional consequences of high HP1 levels differ between inserted transgenes and endogenous Chromosome 4 genes. We speculate that POF is needed for expression of these Chromosome 4 genes and that inserted transgenes will be repressed by HP1 but will fail to recruit POF.

## Materials and Methods

### Chromatin preparation and immunoprecipitation.

For our ChIP experiments we used Schneider's *Drosophila* line 2 cells (ATCC CRL-1963) grown at 25 °C in Erlenmeyer flasks at a density of 0.5–1.5 × 10^7^ cells/ml in *Drosophila* SFM medium (Invitrogen) supplemented with 100 U/ml of Penicillin G, 100 μg/ml of Streptomycin sulfate, and 2 mM of L-glutamine. The cells were cross-linked, washed, and sonicated as described by [51]. For salivary glands, we used 250 pairs of glands for each biological replicate. The glands were dissected in PBS and directly transferred to 2% formaldehyde in PBS with 0.1% Triton X-100 for 1–2 min for fixation. The glands were then washed in PBS and glycine was added to a final concentration of 0.125 M. The glands were then washed once in PBS, 1 mM PMSF, protease inhibitor cocktail (Roche) followed by two washes in TBS, 1 mM PMSF, and protease inhibitor cocktail. The samples were homogenized in 500 μl lysis buffer (1% SDS, 10 mM EDTA, 50 mM Tris-HCl [pH 8.1]). Approximately 120 μl glass beads were added prior to sonication for 2 × 10 sec at output level 1, 2 × 10 sec at output level 2, 2 × 10 sec at output level 3 and finally 2 × 10 s at output level 4 (Misonix XL2020, microtip). The samples were cleared by centrifuging for 10 min at 16,000 g. For ChIP, 150 μl of cell lysate was diluted by a factor of ten in ChIP Dilution buffer (0.01% SDS, 1.1% Triton X-100, 1.2 mM EDTA, 16.7 mM Tris-HCl [pH 8.0], 167 mM NaCl), and protein inhibitors were added. The diluted lysate was precleared by incubation with 30 μl (60 μl for salivary glands) of Dynabeads conjugated to Protein A (Dynal) and preblocked by equilibration in 150 μl ChIP buffer containing 12 μg (24 μg for salivary glands) sonicated herring sperm DNA. The cleared lysates were then incubated with 3 μl anti-POF (rabbit, 4 μl for salivary glands), 3 μl anti-HP1 (291C, Covance, 4 μl for salivary glands), or 4 μl H3K9ac (07–352, Upstate) antibodies overnight at 4 °C. The antibody complexes were precipitated by incubation with DNA-blocked Protein A Dynabeads for 1 h at 4 °C. The beads were washed once with low salt buffer (0.1% SDS, 1% Triton X-100, 2 mM Tris-HCl [pH 8.0], 150 mM NaCl), once with high salt buffer (0.1% SDS, 1% Triton X-100, 2 mM Tris-HCl [pH 8.0], 500 mM NaCl), once with LiCl-containing buffer (250 mM LiCl, 10 mM Tris-HCl [pH 8.0], 1 mM EDTA, 1% NP-40, 1% sodium deoxycholate), and twice with TE Buffer (10 mM Tris-HCl [pH 8.0], 1 mM EDTA). The protein/DNA complexes were eluted from the antibodies by incubating them for 2 × 15 min at room temperature in 250 μl Elution buffer (1% SDS, 0.1 M NaHCO_3_) with rotation. NaCl was added to a final concentration of 200 mM, and protein/DNA crosslinks were reversed by heating at 65 °C for 4 h. A total of 10 μl of 0.5 M EDTA, 20 μl of 1M Tris-HCl [pH 6.5], and 1 μl of 20 mg/ml proteinase K were added before an additional incubation at 45 °C for 1 h. The DNA was recovered by phenol/chloroform extraction followed by ethanol precipitation. The immunoprecipitated DNA was then dissolved in 20 μl water.

### Ligation-mediated PCR.

The immunoprecipitated DNA and corresponding amounts of input DNA were amplified using ligation-mediated PCR. The linkers used and the ligation procedure were as described [52]. The amplification mainly followed [53], except that we ligated and amplified all immunoprecipitated DNA (to retain as much sample complexity as possible) from each sample. We used an Advantage cDNA PCR kit (BD Biosciences) for a 26-cycle PCR amplification. The DNA was purified using illustra DNA and a Gel band purification kit (GE Healthcare) prior to labeling. To verify that no amplification bias affected the enrichment profiles, we analyzed the ChIP DNA/input DNA ratio before and after the ligation-mediated PCR, using real-time PCR as described previously [18].

### Tiling array analysis.

Tiling arrays containing the complete sequenced part of the fourth chromosome at a resolution of 10 bp and of region 1–12,000 kb from Chromosome 2L at a resolution of 50 bp were designed on the basis of D. melanogaster genomic release 4. Repeated regions were removed from the design using a repeat masker. Three pseudogenes (*CR32011*, *CR32010*, and *CR32009*) are present on the fourth chromosome. Because of high sequence identity between these three genes they were not included in further analysis. Thus, 89 genes on the fourth chromosome were included in the analysis. The array production, probe labeling, and hybridization were conducted by Nimblegen Systems Inc. (http://www.nimblegen.com). In total, 15 ChIP experiments and four cDNA samples for transcript profiling were hybridized. The complete dataset is available at http://www.ncbi.nlm.nih.gov/geo/. The raw enrichment data (ChIP/input) were very consistent between the three ChIP replicates and between consecutive probes (Figure S1). We therefore applied a mild smoothing algorithm to process the ChIP-chip data and remove noise but retain as much information as possible. The smoothing was conducted using a seven-probe sliding window approach, in which the median score of the seven probes was assigned to the middle position. The smoothing was undertaken to keep the borders between masked and unmasked regions intact. A five-probe sliding window was used for Chromosome 2L. For HP1 and POF enrichments, cutoff values for binding were calculated as follows. Region 10,600 kb–11,600 kb from 2L was used as a control region to calculate the mean enrichment and standard deviation for each experiment. This region was chosen since it reflects only the background enrichments of POF and HP1. This was also verified for HP1 by the data presented in Greil et al. [54]. All probes (after smoothing) below the mean enrichment + 2 standard deviations level were set to zero. Within each experiment the replicate with the lowest standard deviation (in the control region) was selected for further analysis. All subsequent calculations and evaluations were based on these datasets. Relative binding levels of POF and HP1 for each gene in S2 cells and salivary glands were calculated as the mean enrichment of the half of the probes that were most strongly bound within annotated exons (release 4.3). Genes for which more than half of the respective probes were unbound (as defined above) within exons were deemed to be unbound and their binding levels were set to zero. To verify that our conclusions would not change as a result of the replicate used, we calculated relative gene binding levels for all replicates; pairs of replicates were then compared (Figure S2).

For transcript profile analysis, raw data for each probe were returned from Nimblegen. To remove the background signal a cutoff was calculated as the mean + one standard deviation hybridization signal from the 10,000 random probes present on the arrays (GC content 50%). All values below these cutoffs were set to zero. A relative gene transcription level was set as the mean enrichment of the 50% highest probes within exons of each gene (Table S1). To calculate binding and transcription differences between S2 cells and salivary glands the transcription and binding levels were adjusted so that the total binding and transcription, respectively, were equal in the S2 cells and salivary gland preparations. Details of all algorithms and software used are available upon request.

### Transcript profiling.

For transcript profiling experiments, poly(A)^+^RNA was isolated using DynabeadsOligo (dT)_25_ (Dynal). Two biological replicates were used of both salivary glands (300 pairs per replicate) and S2 cells. The cells or tissue were frozen at −70 °C and homogenized in 0.1 M TRIS-HCl (pH 8.0), 0.5 M LiCl, 10 mM EDTA, 1% SDS, and 5 mM DTT. Dynal's recommendations were then followed. The poly(A)^+^RNA was converted to cDNA using an ImPromII first strand synthesis kit (Promega) according to the supplier's recommendations. The cDNA was then used as a template for forming double-stranded cDNA using a Superscript double stranded cDNA synthesis kit (Invitrogen). The double-stranded cDNA was purified by phenol-chloroform extraction followed by passage through S200HR columns (GE Healthcare), and 4 μg of the double-stranded cDNA from each sample was sent to Nimblegen for labeling and hybridization using the same array design as for the ChIP-chip.

### Expression and HP1 binding surrounding transgene insertions.

To determine expression (transcript levels) and HP1 enrichment close to *P* element insertions on the fourth chromosome, we used the data from Sun et al. [35] and their classification as variegated (*n* = 17) or nonvariegated (*n* = 6). The highest relative gene expression value and relative gene HP1 enrichment levels (calculated as described above) within 1 kb (±500 bp) were determined for each insertion, then mean expression and HP1 enrichment levels were calculated.

## Supporting Information

Figure S1 The ChIP DNAs Were Checked before and after Ligation-Mediated PCR and before Labeling (A) POF enrichment before and after ligation-mediated PCR as determined by real-time PCR.
(B) POF enrichments in a randomly chosen 10-kb region (1,225–1,235 kb) from the three biological replicates of S2 cells. The effect of the seven-probe median smoothing process is shown below the raw data from the replicates.
(C) The enrichment profile in the 216–227-kb genomic region from Johansson et al. [18] compared to the enrichment profile determined using the ChIP-chip. Note that the 222-kb region is masked on the array.
(D) The two HP1 antibodies (C1A9 and PRB291C) give comparable enrichment profiles. PRB291C was used in the ChIP-chip experiments. The *y*-axis represents % of input as determined by real-time PCR.
(134 KB PDF)

Figure S2 Gene Binding Values for POF and HP1 The gene binding values for POF and HP1 were individually calculated from each of the three replicates of POF in S2 Cells (PS2), POF in Salivary Glands (PSG), HP1 in S2 Cells (HS2), and HP1 in Salivary Glands (HSG). Pairs of replicates were then compared. Binding values for: POF to genes in S2 cells are plotted in (A–C); POF to genes in salivary glands in (D–F), HP1 to genes in S2 cells in (G–I), and HP1 to genes in salivary glands in (J–L). The replicates used for further analysis are indicated by asterisks.
(106 KB PDF)

Figure S3 Relative Transcription Values for Chromosome 4 Genes in S2 Cells and Salivary Glands Relative transcription values for Chromosome 4 genes in S2 cells and salivary glands were individually calculated from data acquired from each of the two replicates of S2 cells (TP_S2) and salivary glands (TP_SG). Pairs of replicates were then compared, using correlation plots. The replicates used for further analysis are indicated by asterisks.
(42 KB PDF)

Figure S4 There Is a Positive Correlation between the Differences in Transcript Levels in S2 Cells and Salivary Glands (*y*-Axis) and POF Binding Differences (*x*-Axis) The plot shows normalized log2 differences in transcript levels and POF binding, respectively, between S2 cells and salivary glands.
(63 KB PDF)

Table S1 Calculated Relative POF and HP1 Binding Values and Transcription Values for Chromosome 4 Genes in S2 Cells and Salivary Glands (29 KB XLS)

### Accession Numbers

The complete dataset mentioned in this paper is available from the National Center for Biotechnology Information (NCBI) (http://www.ncbi.nlm.nih.gov/geo) (accession number GSE8301).

## Abbreviations

ChIP: chromatin immunoprecipitation
H3K9ac: histone 3 acetylated at lysine 9
H3K9me2/3: di- and tri-methylated histone 3 lysine 9
HP1: heterochromatin protein 1
MSL: male-specific lethal
PEV: position-effect variegation
POF: Painting of Fourth

## References

[pgen-0030209-b001] Grewal SI, Elgin SC (2002). Heterochromatin: new possibilities for the inheritance of structure. Curr Opin Genet Dev.

[pgen-0030209-b002] Ebert A, Lein S, Schotta G, Reuter G (2006). Histone modification and the control of heterochromatic gene silencing in *Drosophila*. Chromosome Res.

[pgen-0030209-b003] Riddle NC, Elgin SC (2006). The dot chromosome of *Drosophila*: insights into chromatin states and their change over evolutionary time. Chromosome Res.

[pgen-0030209-b004] Locke J, McDermid H (1993). Analysis of *Drosophila* chromosome four by pulse field electrophoresis. Chromosoma.

[pgen-0030209-b005] Barigozzi C, Dolfini S, Fraccaro M, Raimondi GR, Tiepolo L (1966). In vitro study of the DNA replication patterns of somatic chromosomes of Drosophila melanogaster. Exp Cell Res.

[pgen-0030209-b006] Ashburner M, Golic KG, Hawley RS (2005). *Drosophila* A laboratory handbook.

[pgen-0030209-b007] Hochman B, Ashburner M, Novitski E (1976). The fourth chromosome of Drosophila melanogaster. The Genetics and biology of *Drosophila*.

[pgen-0030209-b008] Sandler L, Szauter P (1978). The effect of recombination-defective meiotic mutants on fourth-chromosome crossing over in Drosophila melanogaster. Genetics.

[pgen-0030209-b009] Kaminker JS, Bergman CM, Kronmiller B, Carlson J, Svirskas R (2002). The transposable elements of the Drosophila melanogaster euchromatin: a genomics perspective. Genome Biol.

[pgen-0030209-b010] Locke J, Howard LT, Aippersbach N, Podemski L, Hodgetts RB (1999). The characterization of *DINE-1*, a short, interspersed repetitive element present on chromosome and in the centric heterochromatin of Drosophila melanogaster. Chromosoma.

[pgen-0030209-b011] Locke J, Podemski L, Roy K, Pilgrim D, Hodgetts R (1999). Analysis of two cosmid clones from chromosome 4 of Drosophila melanogaster reveals two new genes amid an unusual arrangement of repeated sequences. Genome Res.

[pgen-0030209-b012] Miklos GLG, Yamamoto MT, Davies J, Pirrotta V (1988). Microcloning reveals a high frequency of repetitive sequences characteristic of chromosome four and the beta-heterochromatin of Drosophila melanogaster. Proc Natl Acad Sci U S A.

[pgen-0030209-b013] Pimpinelli S, Berloco M, Fanti L, Dimitri P, Bonaccorsi S (1995). Transposable elements are stable structural components of Drosophila melanogaster heterochromatin. Proc Natl Acad Sci U S A.

[pgen-0030209-b014] Stenberg P, Pettersson F, Saura AO, Berglund A, Larsson J (2005). Sequence analysis of chromosome identity in three *Drosophila* species. BMC Bioinformatics.

[pgen-0030209-b015] Sun FL, Cuaycong MH, Craig CA, Wallrath LL, Locke J (2000). The fourth chromosome of Drosophila melanogaster: interspersed euchromatic and heterochromatic domains. Proc Natl Acad Sci U S A.

[pgen-0030209-b016] Wallrath L, Elgin S (1995). Position effect variegation in *Drosophila* is associated with an altered chromatin structure. Genes Dev.

[pgen-0030209-b017] Wallrath L, Guntur V, Rosman L, Elgin S (1996). DNA representation of variegating heterochromatic P-element inserts in diploid and polytene tissues of Drosophila melanogaster. Chromosoma.

[pgen-0030209-b018] Johansson AM, Stenberg P, Bernhardsson C, Larsson J (2007). Painting of fourth and chromosome-wide regulation of the 4th chromosome in Drosophila melanogaster. EMBO J.

[pgen-0030209-b019] Larsson J, Chen JD, Rasheva V, Rasmuson Lestander A, Pirrotta V (2001). Painting of fourth, a chromosome-specific protein in *Drosophila*. Proc Natl Acad Sci U S A.

[pgen-0030209-b020] Larsson J, Meller VH (2006). Dosage compensation, the origin and the afterlife of sex chromosomes. Chromosome Res.

[pgen-0030209-b021] Larsson J, Svensson MJ, Stenberg P, Mäkitalo M (2004). Painting of fourth in genus *Drosophila* suggests autosome-specific gene regulation. Proc Natl Acad Sci U S A.

[pgen-0030209-b022] Bannister AJ, Zegerman P, Partridge JF, Miska EA, Thomas JO (2001). Selective recognition of methylated lysine 9 on histone H3 by the HP1 chromo domain. Nature.

[pgen-0030209-b023] Jacobs SA, Khorasanizadeh S (2002). Structure of HP1 chromodomain bound to a lysine 9-methylated histone H3 tail. Science.

[pgen-0030209-b024] Nielsen PR, Nietlispach D, Mott HR, Callaghan J, Bannister A (2002). Structure of the HP1 chromodomain bound to histone H3 methylated at lysine 9. Nature.

[pgen-0030209-b025] Czermin B, Melfi R, McCabe D, Seitz V, Imhof A (2002). *Drosophila* enhancer of Zeste/ESC complexes have a histone H3 methyltransferase activity that marks chromosomal Polycomb sites. Cell.

[pgen-0030209-b026] Schotta G, Ebert A, Krauss V, Fischer A, Hoffmann J (2002). Central role of *Drosophila* SU(VAR)3–9 in histone H3-K9 methylation and heterochromatic gene silencing. EMBO J.

[pgen-0030209-b027] Seum C, Reo E, Peng H, Rauscher FJ, Spierer P (2007). *Drosophila* SETDB1 is required for Chromosome 4 silencing. PLoS Genet.

[pgen-0030209-b028] Tzeng TY, Lee CH, Chan LW, Shen CK (2007). Epigenetic regulation of the *Drosophila* chromosome 4 by the histone H3K9 methyltransferase dSETDB1. Proc Natl Acad Sci U S A.

[pgen-0030209-b029] Fanti L, Berloco M, Piacentini L, Pimpinelli S (2003). Chromosomal distribution of heterochromatin protein 1 (HP1) in *Drosophila*: a cytological map of euchromatic HP1 binding sites. Genetica.

[pgen-0030209-b030] James TC, Eissenberg JC, Craig C, Dietrich V, Hobson A (1989). Distribution patterns of HP1, a heterochromatin-associated nonhistone chromosomal protein of *Drosophila*. Eur J Cell Biol.

[pgen-0030209-b031] Koryakov DE, Reuter G, Dimitri P, Zhimulev IF (2006). The *SuUR* gene influences the distribution of heterochromatic proteins HP1 and SU(VAR)3–9 on nurse cell polytene chromosomes of Drosophila melanogaster. Chromosoma.

[pgen-0030209-b032] de Wit E, Greil F, van Steensel B (2007). High-resolution mapping reveals links of HP1 with active and inactive chromatin components. PLoS Genet.

[pgen-0030209-b033] de Wit E, Greil F, van Steensel B (2005). Genome-wide HP1 binding in *Drosophila*: developmental plasticity and genomic targeting signals. Genome Res.

[pgen-0030209-b034] Haynes KA, Caudy AA, Collins L, Elgin SC (2006). Element *1360* and RNAi components contribute to HP1-dependent silencing of a pericentric reporter. Curr Biol.

[pgen-0030209-b035] Sun FL, Haynes K, Simpson CL, Lee SD, Collins L (2004). *cis*-Acting determinants of heterochromatin formation on Drosophila melanogaster chromosome four. Mol Cell Biol.

[pgen-0030209-b036] Batsché E, Yaniv M, Muchardt C (2006). The human SWI/SNF subunit Brm is a regulator of alternative splicing. Nat Struct Mol Biol.

[pgen-0030209-b037] Pokholok DK, Harbison CT, Levine S, Cole M, Hannett NM (2005). Genome-wide map of nucleosome acetylation and methylation in yeast. Cell.

[pgen-0030209-b038] Ayyanathan K, Lechner MS, Bell P, Maul GG, Schultz DC (2003). Regulated recruitment of HP1 to a euchromatic gene induces mitotically heritable, epigenetic gene silencing: a mammalian cell culture model of gene variegation. Genes Dev.

[pgen-0030209-b039] Feldman N, Gerson A, Fang J, Li E, Zhang Y (2006). G9a-mediated irreversible epigenetic inactivation of Oct-3/4 during early embryogenesis. Nat Cell Biol.

[pgen-0030209-b040] Nielsen SJ, Schneider R, Bauer UM, Bannister AJ, Morrison A (2001). Rb targets histone H3 methylation and HP1 to promoters. Nature.

[pgen-0030209-b041] Young AP, Longmore GD (2004). Differences in stability of repressor complexes at promoters underlie distinct roles for Rb family members. Oncogene.

[pgen-0030209-b042] Li B, Carey M, Workman JL (2007). The role of chromatin during transcription. Cell.

[pgen-0030209-b043] Clegg NJ, Honda BM, Whitehead IP, Grigliatti TA, Wakimoto B (1998). Suppressors of position-effect variegation in Drosophila melanogaster affect expression of the heterochromatic gene *light* in the absence of a chromosome rearrangement. Genome.

[pgen-0030209-b044] Hearn MG, Hedrick A, Grigliatti TA, Wakimoto BT (1991). The effect of modifiers of position-effect variegation on the variegation of heterochromatic genes of Drosophila melanogaster. Genetics.

[pgen-0030209-b045] Lu BY, Emtage PC, Duyf BJ, Hilliker AJ, Eissenberg JC (2000). Heterochromatin protein 1 is required for the normal expression of two heterochromatin genes in *Drosophila*. Genetics.

[pgen-0030209-b046] Piacentini L, Fanti L, Berloco M, Perrini B, Pimpinelli S (2003). Heterochromatin protein 1 (HP1) is associated with induced gene expression in *Drosophila* euchromatin. J Cell Biol.

[pgen-0030209-b047] Vakoc CR, Mandat SA, Olenchock BA, Blobel GA (2005). Histone H3 lysine 9 methylation and HP1gamma are associated with transcription elongation through mammalian chromatin. Mol Cell.

[pgen-0030209-b048] Alekseyenko AA, Larschan E, Lai WR, Park PJ, Kuroda MI (2006). High-resolution ChIP-chip analysis reveals that the *Drosophila* MSL complex selectively identifies active genes on the male X chromosome. Genes Dev.

[pgen-0030209-b049] Gilfillan GD, Straub T, de Wit E, Greil F, Lamm R (2006). Chromosome-wide gene-specific targeting of the *Drosophila* dosage compensation complex. Genes Dev.

[pgen-0030209-b050] Legube G, McWeeney SK, Lercher MJ, Akhtar A (2006). X-chromosome-wide profiling of MSL-1 distribution and dosage compensation in *Drosophila*. Genes Dev.

[pgen-0030209-b051] Schwartz YB, Kahn TG, Nix DA, Li XY, Bourgon R (2006). Genome-wide analysis of Polycomb targets in Drosophila melanogaster. Nat Genet.

[pgen-0030209-b052] Strutt H, Cavalli G, Paro R (1997). Co-localization of Polycomb protein and GAGA factor on regulatory elements responsible for the maintenance of homeotic gene expression. EMBO J.

[pgen-0030209-b053] Ren B, Dynlacht BD (2004). Use of chromatin immunoprecipitation assays in genome-wide location analysis of mammalian transcription factors. Methods Enzymol.

[pgen-0030209-b054] Greil F, van der Kraan I, Delrow J, Smothers JF, de Wit E (2003). Distinct HP1 and Su(var)3–9 complexes bind to sets of developmentally coexpressed genes depending on chromosomal location. Genes Dev.

