# Supplementary material for: POF and HP1 Bind Expressed Exons, Suggesting a Balancing Mechanism for Gene Regulation
Source: PLoS Genet. 2007 Nov 16;3(11):e209. doi: 10.1371/journal.pgen.0030209 (PMC2077892; doi:10.1371/journal.pgen.0030209)
Supplement: Supplementary file 1 [file pgen.0030209.sg001.pdf]

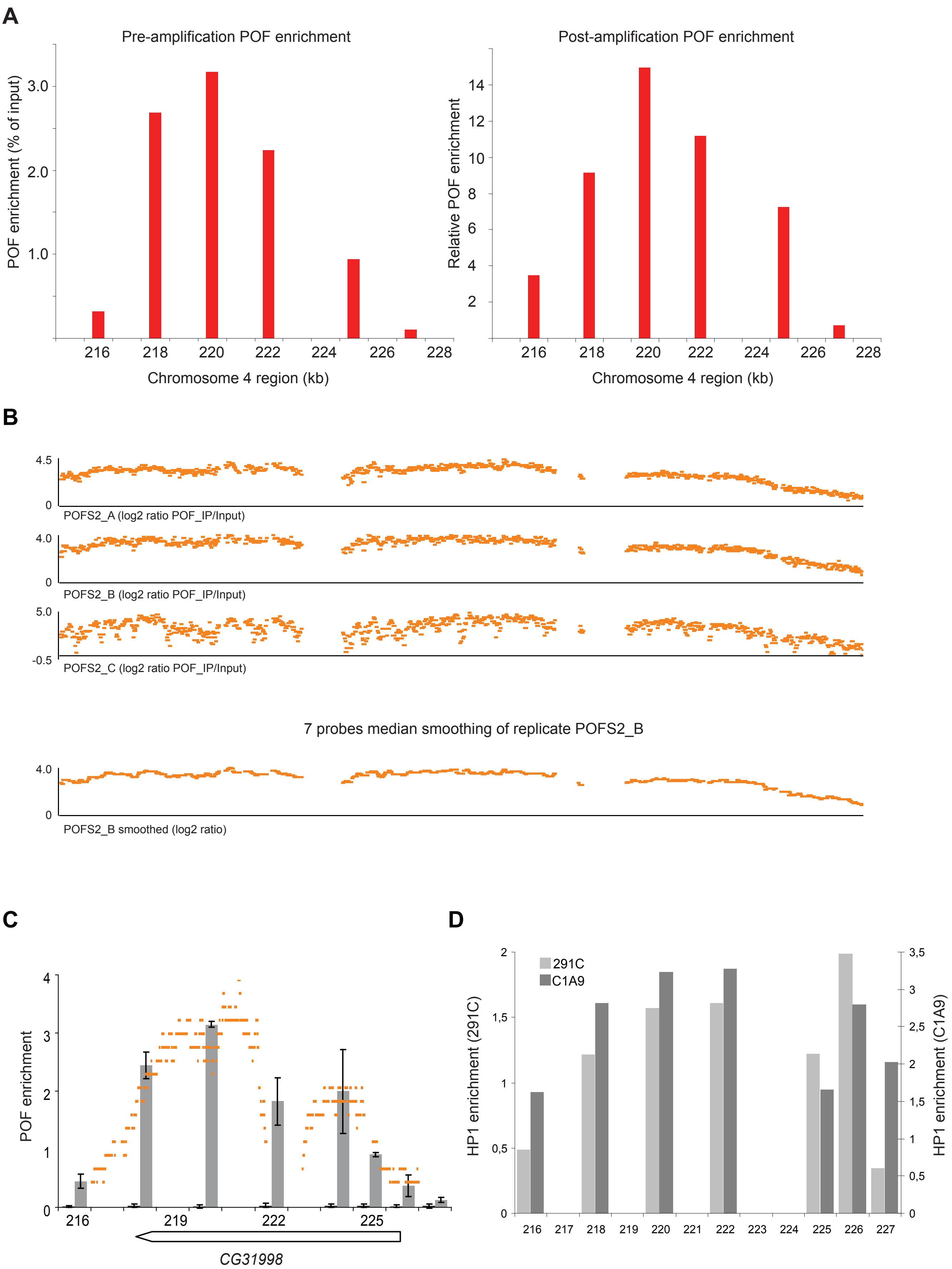
A
Pre-amplification POF enrichment
POF enrichment (% of input)
3.0
2.0
1.0
216
218
220
222
224
226
228
Chromosome 4 region (kb)
Post-amplification POF enrichment
Relative POF enrichment
14
12
10
8
6
4
2
216
218
220
222
224
226
228
Chromosome 4 region (kb)
B
4.5
0
POFS2_A (log2 ratio POF_IP/Input)
4.0
0
POFS2_B (log2 ratio POF_IP/Input)
5.0
-0.5
POFS2_C (log2 ratio POF_IP/Input)
7 probes median smoothing of replicate POFS2_B
4.0
0
POFS2_B smoothed (log2 ratio)
C
POF enrichment
4
3
2
1
0
216
219
222
225
CG31998
D
291C
C1A9
HP1 enrichment (291C)
2
1,5
1
0,5
0
HP1 enrichment (C1A9)
3,5
3
2,5
2
1,5
1
0,5
0
216
217
218
219
220
221
222
223
224
225
226
227
