# Supplementary material for: POF and HP1 Bind Expressed Exons, Suggesting a Balancing Mechanism for Gene Regulation
Source: PLoS Genet. 2007 Nov 16;3(11):e209. doi: 10.1371/journal.pgen.0030209 (PMC2077892; doi:10.1371/journal.pgen.0030209)

Johansson_FigS4

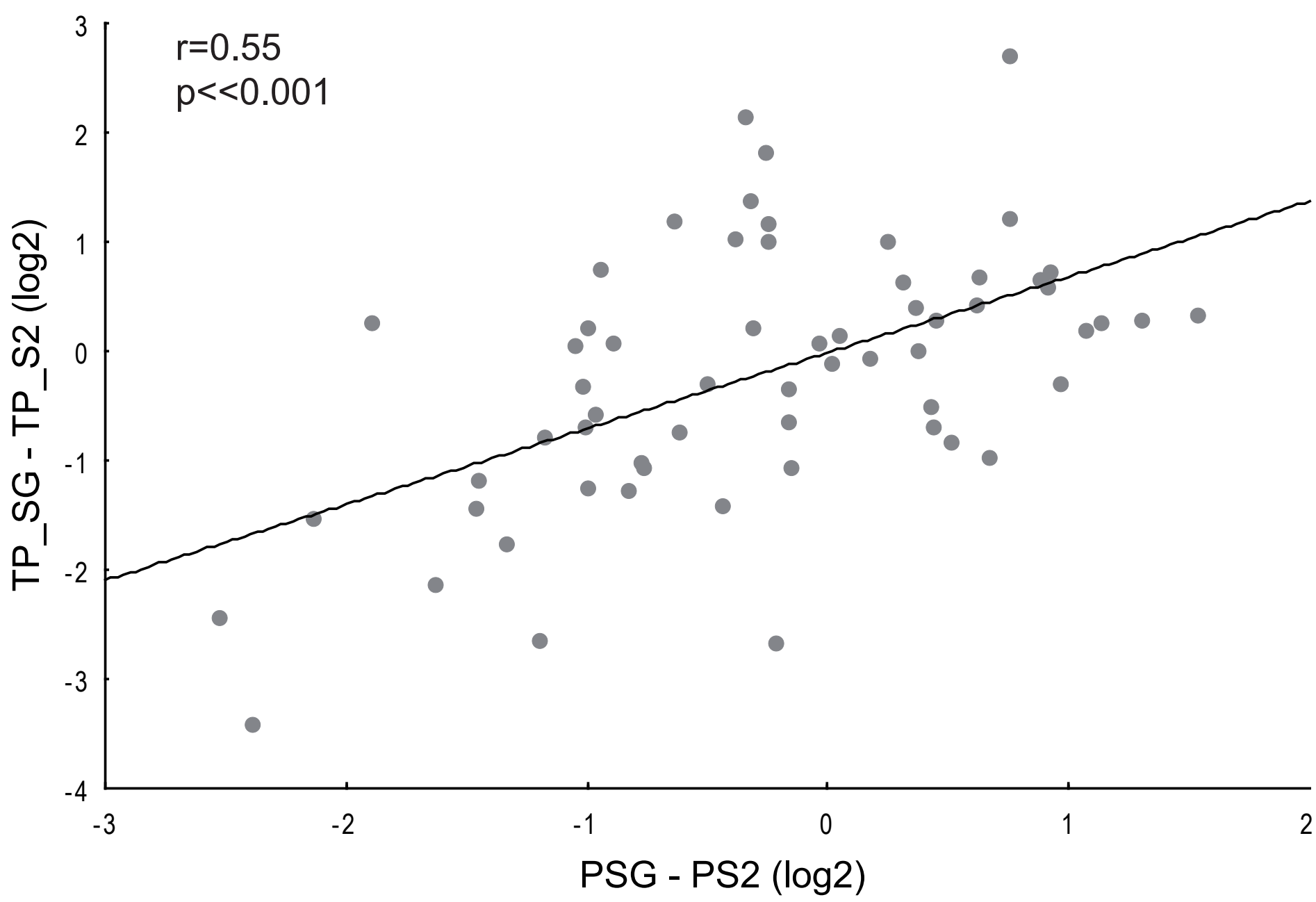

Supplement: Supplementary file 4 [file pgen.0030209.sg004.pdf]
